# Characteristics of fetuses evaluated due to suspected anencephaly: a population-based cohort study in southern Brazil: Características de fetos avaliados por suspeita de anencefalia: um estudo de coorte de base populacional do Sul do Brasil

**DOI:** 10.1590/1516-3180.2013.8012608

**Published:** 2015-03-17

**Authors:** Emanuele Pelizzari, Carolina Melendez Valdez, Jamile dos Santos Picetti, André Campos da Cunha, Cristine Dietrich, Paulo Renato Krahl Fell, Luciano Vieira Targa, Paulo Ricardo Gazzola Zen, Rafael Fabiano Machado Rosa

**Affiliations:** I MD. Physician, Residency Program in Obstetrics and Gynecology, Hospital Materno Infantil Presidente Vargas (HMIPV), Porto Alegre, Rio Grande do Sul, Brazil.; II MD. Physician, Residency Program in Fetal Medicine, Hospital Materno Infantil Presidente Vargas (HMIPV), Porto Alegre, Rio Grande do Sul, Brazil.; III MD. Obstetrician, Fetal Medicine, Hospital Materno Infantil Presidente Vargas (HMIPV), Porto Alegre, Rio Grande do Sul, Brazil.; IV MD. Pediatric Radiologist, Hospital Materno Infantil Presidente Vargas (HMIPV), Porto Alegre, Rio Grande do Sul, Brazil.; V PhD. Adjunct Professor of Clinical Genetics and of the Postgraduate Program on Pathology, Universidade Federal de Ciências da Saúde de Porto Alegre (UFCSPA), and Clinical Geneticist, Universidade Federal de Ciências da Saúde de Porto Alegre (UFCSPA) and Complexo Hospitalar Santa Casa de Porto Alegre (CHSCPA), Porto Alegre, Rio Grande do Sul, Brazil.; VI PhD. Clinical Geneticist, Universidade Federal de Ciências da Saúde de Porto Alegre (UFCSPA), Complexo Hospitalar Santa Casa de Porto Alegre (CHSCPA) and Hospital Materno Infantil Presidente Vargas (HMIPV), Porto Alegre, Rio Grande do Sul, Brazil.

**Keywords:** Anencephaly, Neural tube, Amniotic band syndrome, Ultrasonics, Genetic counseling

## Abstract

**CONTEXT AND OBJECTIVE:**

Anencephaly is considered to be the most common type of neural tube defect. Our aim was to assess the clinical and gestational features of a cohort of fetuses with suspected anencephaly.

**DESIGN AND SETTING:**

Population-based retrospective cohort study in a referral hospital in southern Brazil.

**METHODS:**

The sample consisted of fetuses referred due to suspected anencephaly, to the Fetal Medicine Service of Hospital Materno Infantil Presidente Vargas, between January 2005 and September 2013. Clinical, radiological, pathological and survival data were gathered.

**RESULTS:**

Our sample was composed of 29 fetuses. The diagnosis of suspected anencephaly was made on average at 21.3 weeks of gestation. Seven fetuses had malformations that affected other organs, and these included oral clefts (n = 4) and congenital heart defects (n = 2). In 16 cases, there was termination of pregnancy (n = 12) or intrauterine death (n = 4). Regarding those who were born alive (n = 13), all of them died in the first week of life. After postnatal evaluation, the diagnosis of anencephaly was confirmed in 22 cases (75.9%). Other conditions included amniotic band disruption complex (6.9%), microhydranencephaly (6.9%), merocrania (3.4%) and holoprosencephaly (3.4%).

**CONCLUSIONS:**

Different conditions involving the cranial vault may be confused with anencephaly, as seen in our sample. However, these conditions also seem to have a poor prognosis. It seems that folic acid supplementation is not being properly performed.

## RESUMO

**CONTEXTO E OBJETIVO:**

A anencefalia é considerada o tipo mais comum de defeito de fechamento do tubo neural. Nosso objetivo foi avaliar as características clínicas e gestacionais de uma coorte de fetos com suspeita de anencefalia.

**TIPO DE ESTUDO E LOCAL:**

Estudo de coorte retrospectivo de base populacional em um hospital de referência no sul do Brasil.

**MÉTODOS:**

A amostra foi composta por fetos encaminhados por suspeita de anencefalia ao Serviço de Medicina Fetal do Hospital Materno Infantil Presidente Vargas, no período de janeiro de 2005 a setembro de 2013. Foi realizada coleta de dados clínicos, radiológicos, patológicos e de sobrevida.

**RESULTADOS:**

Nossa amostra foi composta por 29 fetos. A suspeita do diagnóstico de anencefalia foi realizada em média com 21,3 semanas de gestação. Sete fetos apresentavam malformações que afetavam outros órgãos, e incluíram fendas orais (n = 4) e defeitos cardíacos congênitos (n = 2). Em 16 casos houve interrupção da gravidez (n = 12) ou morte intrauterina (n = 4). Daqueles que nasceram vivos (n = 13), todos morreram na primeira semana de vida. Após a avaliação pós-natal, o diagnóstico de anencefalia foi confirmado em 22 casos (75,9%). Outras condições incluíram o complexo disruptivo de banda amniótica (6,9%), microhidranencefalia (6,9%), merocrania (3,4%) e holoprosencefalia (3,4%).

**CONCLUSÕES:**

Diferentes condições que envolvem a calota craniana podem ser confundidas com a anencefalia, como verificado em nossa amostra. No entanto, estas também parecem ter um prognóstico pobre. A suplementação com ácido fólico parece não estar sendo realizada de forma adequada.

## INTRODUCTION

Neural tube defects are considered to be one of the most common malformations observed at birth. Among these is anencephaly, which is the most common type, occurring in about 1 in every 1,000 births in the United States and 5 in every 1,000 births in Ireland and Wales.^1^^,^^2^^,^^3^ It is considered to be a severe malformation of the central nervous system, and its prenatal diagnosis through ultrasound has been possible for over 40 years.^1^^,^^4^

The etiology of anencephaly is attributed to a failure of neurulation that may be associated with genetic or environmental factors, or both.^1^ Furthermore, it may be difficult to differentiate anencephaly from other conditions with involvement of the cranial vault during the prenatal period, and this may have important implications for pregnancy management (including determination of the prognosis and legal issues) and for the genetic counseling to be given to the family. Furthermore, there is a paucity of studies evaluating these matters, not only in the Brazilian literature but also in the worldwide literature.^5^^,^^6^^,^^7^

## OBJECTIVE

Our aim was to assess the clinical and gestational features of a cohort of fetuses with suspected anencephaly.

## METHODS

This was a population-based retrospective cohort study in a referral hospital in southern Brazil. The sample consisted of fetuses referred due to suspected anencephaly, to the Fetal Medicine Service of Hospital Materno Infantil Presidente Vargas (HMIPV), between January 2005 and September 2013. We gathered clinical, radiological, pathological and survival data from the medical records. This project was approved by the Research Ethics Committees of our university and hospital. The data retrieved from the medical records consisted of maternal age and ethnicity; history of parental consanguinity; origin; maternal occupation; data on obstetric past; medication use (including folic acid); exposure to tobacco, alcohol and illicit drugs; maternal diseases; obstetric complications; family history of neural tube defects; ultrasound examinations performed during pregnancy, noting how many were done and at what stage, and describing the results; additional tests such as echocardiography, magnetic resonance imaging and karyotyping; data relating to performing legal termination of pregnancy; occurrence of intrauterine death and postnatal survival; delivery and perinatal data; autopsy results; and final diagnosis. The final diagnosis was reviewed in all cases, based on the findings obtained through the research protocol and the images from the hospital’s database. Gestational age was determined according to the earlier ultrasound. We excluded fetuses that were lost from the prenatal follow-up at our service or had incomplete medical records.

We used the PEPI software for Windows and the Statistical Package for the Social Sciences (SPSS), version 12.0 for Windows, to analyze the results. The tests applied were the two-tailed Fisher exact test for comparisons of frequencies and the t test for comparisons of means. P values < 0.05 were considered to be significant. The Kaplan-Meier test was used through the BioEstat 5.0 software, to construct pregnancy termination and survival curves.

## RESULTS

Over a period of about 9 years, 32 fetuses with suspected anencephaly were identified. Among these fetuses, three were excluded because they were lost from the prenatal follow-up (n = 1) or had incomplete medical records (n = 2). Thus, our final sample was composed of 29 fetuses. The demographic data relating to this sample can be seen in Table 1. In only eight cases, there was a note in the medical record regarding consanguinity and all patients were found to be non-consanguineous. Regarding origin, the majority of the fetuses came from areas peripheral to Porto Alegre (the city where the hospital is located) (41.8%); nine (31%) were referred from within the city and eight (27.2%) came from other municipalities in the state of Rio Grande do Sul. Regarding maternal occupation, the majority were housewives (44.8%). Two pregnant women (6.9%) had a history of working with chemical agents.

**Table 1.**
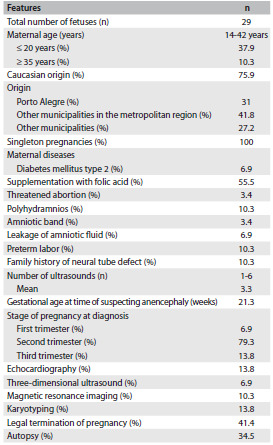
Demographic data of the sample

All cases in the sample consisted of singleton pregnancies (there were no twins). The number of pregnancies ranged from one to five (mean: 2.3). Three pregnant women (10.3%) had a history of one previous abortion. Maternal diseases were described in four cases (13.8%) and consisted of diabetes mellitus type 2 (n = 2), gestational diabetes mellitus (n = 1) and congenital cataract (n = 1). Both of the pregnant women with type 2 diabetes mellitus required insulin treatment. Data on the use of folic acid was reported in only nine cases (31%) and in five, supplementation was administered. However, this was done after the second month of pregnancy in all cases. Regarding gestational exposure, five pregnant women (17.2%) reported smoking, three (10.3%) alcohol intake and two (6.9%) use of illicit drugs (one case of cocaine and one of marijuana) (Table 1).

The diagnosis of suspected anencephaly was made on average at a gestational age of 21.3 weeks (ranging from 12 to 34 weeks). The complementary examinations performed can be seen in Table 1. There were no cases of chromosomal abnormalities. Seven fetuses had malformations that affected other organs and systems outside the central nervous system. These included oral clefts (n= 4), congenital heart defects (n = 2), microphthalmia (n = 2), hypertelorism (n = 1), hypotelorism (n = 1) and single umbilical artery (n = 1).

**Figure 1.**
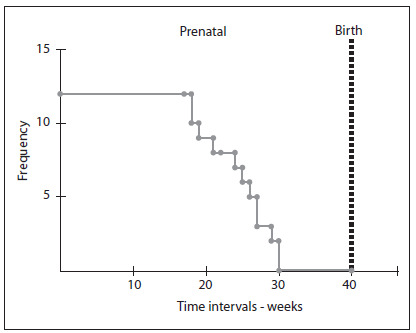
Kaplan-Meier curve showing the times of pregnancy termination in cases that were legally interrupted.

In 12 cases, there was a request for legal termination of pregnancy. The time of discontinuation ranged from 18 to 30 weeks of gestation (mean of 24.3 weeks). The time between completion of the application and interruption ranged from 1 to 15 days (mean of 4.6 days). Only one fetus was evaluated after the official legalization of pregnancy termination due to fetal anencephaly. Four fetuses already presented intrauterine fetal death at the time of interruption (Figure 1). Among the pregnancies that were not interrupted (n = 17), 4 (23.5%) evolved to intrauterine death. The gestational age of these cases ranged from 22 to 40 weeks. Among the pregnancies that were not interrupted and did not evolve to intrauterine death (n = 13), the majority (76.9%) were delivered vaginally, and the gestational age at birth ranged from 17 to 41 weeks (the average was 38.8 weeks, and there were four preterm cases). Birth weight ranged from 183 to 4,380 g (mean 2,392 g). All of these infants died during their first week of life, and 11 of them (84.6%) died on the first day.

**Figure 2.**
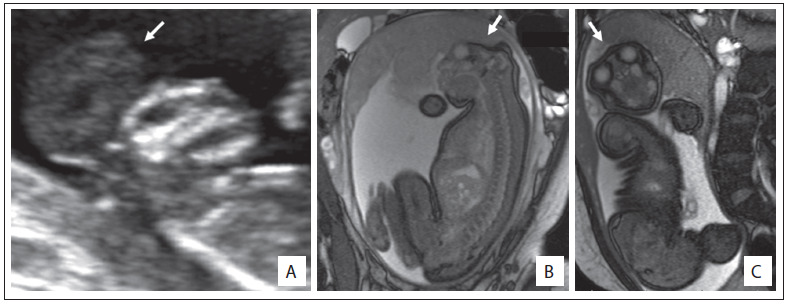
Two-dimensional ultrasound showing the profile of fetuses presenting exencephaly, i.e. the first stage of anencephaly (case 1). Note also the presence of a significant amount of brain tissue (A). Appearance of one of the fetuses (case 12) with anencephaly (see arrows) evaluated through magnetic resonance imaging (B and C).

**Figure 3.**
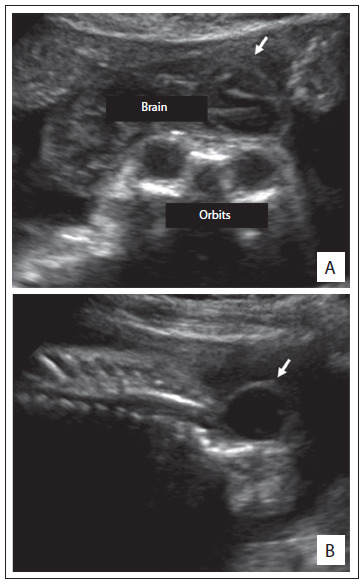
Ultrasound findings from a fetus (case 7) still in at the stage of exencephaly (A, see arrows) and with myelomeningocele (B).

Out of the whole sample, 16 fetuses (55.2%) were male. In 10 cases (34.5%), an autopsy was performed (Table 1). None of the fetuses underwent radiographic evaluation. Interestingly, one of the fetuses with anencephaly was born with the umbilical cord attached to his cephalic pole. In 22 cases (75.9%), the diagnosis of anencephaly was confirmed. Other conditions that were diagnosed included amniotic band disruption complex (6.9%), microhydranencephaly/fetal brain disruption sequence (6.9%), merocrania (3.4%) and holoprosencephaly (3.4%). There was one case without a definitive diagnosis (Table 2 and Figures 2 and 3).

**Table 2.**
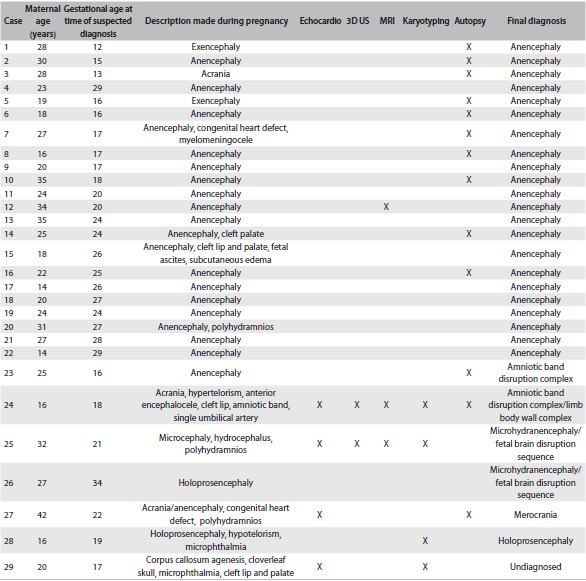
Main findings, assessments and diagnoses found among the 29 fetuses

Echocardio = echocardiography; 3D US = three-dimensional ultrasound; MRI = magnetic resonance imaging.

In comparing the group of fetuses diagnosed with anencephaly (n = 22) with the rest of the sample (n = 7), no significant differences were observed in relation to fetal sex (40.9% versus 57.1% females) (P = 0.6665); mother’s age at the time of pregnancy (the averages were 24.2 years and 25.4 years, respectively); or frequency of primiparity (45.8% versus 71.4%, respectively; P = 0.3898). The timing of prenatal suspicion was also similar (in most cases and in both groups, this occurred during the second trimester of pregnancy: 77.3% versus 85.7%). However, there were two cases with anencephaly (9%) in which the diagnosis was suspected during the first trimester of pregnancy. A family history of neural tube defects was observed only in cases of anencephaly (13.6%).

Most fetuses in both groups who were alive at birth died on the first day of life: all nine of the infants with anencephaly (100%), and four out of the five infants with another diagnosis. The only infant that lived beyond the first day of life was diagnosed with amniotic band disruption complex (presenting acrania; case 24) and survived for four days.

## DISCUSSION

According to the Brazilian live birth information system (SINASC), the frequency of births of individuals with anencephaly in the state of Rio Grande do Sul in 2005 was 32 cases out of 147,199 births, i.e. there was a report of one case per 4,600 live births. This proportion is lower than what has been described in the literature in other countries, such as the United States, as we noted before.^1^ These frequencies may be influenced by termination of the pregnancy in cases of anencephaly, and by the supplementation with folic acid, which started at the beginning of the 2000s in Brazil. However, we believe that this result could be an underestimate.

During the period evaluated in the present study, at the Fetal Medicine Service of HMIPV, there were nine confirmed cases of anencephaly that were born alive (13 were interrupted or presented intrauterine death). This service is a reference in the state of Rio Grande do Sul for pregnant women attended through the Brazilian National Health System (Sistema Único de Saúde, SUS). Thus, many cases were born outside of the hospital, thus maybe indicating that many cases of anencephaly could have been born without this diagnosis having been made during pregnancy. Another point to be considered is the fact that because of the impossibility of treatment and lethality of anencephaly, it is a condition without investment. For this reason, perhaps, many cases prenatally diagnosed in hospitals other than HMIPV were not referred to our service, i.e. were handled locally.

Prenatal ultrasound is able to detect ossified portions of the fetal skeleton from the end of the first trimester of pregnancy onwards. In cases of anencephaly, the unprotected brain tissue undergoes gradual degeneration and destruction due to mechanical and chemical trauma, thereby leading to complete or nearly complete disappearance of the brain around the 14^th^ week of gestation. Thus, at an early stage during the first trimester of pregnancy, there is still a variable amount of brain tissue. Therefore, exencephaly is considered to be the best term to describe anencephaly during this initial stage.^8^

Nowadays, prenatal detection of anencephaly by means of ultrasound is possible in almost 100% of cases. Moreover, during the last decade, it has become possible to diagnose this condition in the early stages of pregnancy: several case series and case reports have been published confirming the possibility of diagnosis from the 10^th^ week of gestation onwards. Anencephaly can be diagnosed by demonstrating the absence of a cranial vault. However, in the first trimester, this can be particularly difficult because of the variable quantity of brain tissue that may be present.^4^^,^^9^ In our sample, two cases of anencephaly were diagnosed during this gestational period. The diagnosis of suspected anencephaly was made on average at a gestational age of 21.3 weeks. We believe that this finding may be related to delay in the arrival of these fetuses for specialized evaluation. In addition, this may have important implications, especially with regard to termination of pregnancy.

Reduction in the crown-rump length or chin-vertex length and increased echogenicity of the amniotic fluid are findings that may assist in confirming the diagnosis of anencephaly.^4^^,^^10^^,^^11^ Surface images can also be obtained by means of three-dimensional ultrasound.^12^ However, in our sample, this approach was little used, as noted in Tables 1 and 2. Magnetic resonance imaging (MRI) can also improve the accuracy of ultrasound when this alone cannot provide all the answers.^13^ In our sample, the applicability of MRI was quite evident, especially for the differential diagnosis (Tables 1 and 2).

Some authors have also been drawing attention to the importance of the postmortem examination and simple radiographic studies for evaluating associated malformations and confirming the prenatal findings. These are straightforward, inexpensive and effective methods.^6^^,^^14^ In our sample, the autopsy was important for defining the correct diagnosis in four cases (Table 2). Unfortunately, none of our fetuses underwent radiographic evaluation after birth.

Some environmental factors are known to be associated with higher chances of occurrences of anencephaly, such as pregestational diabetes and anticonvulsant drugs.^1^ In our sample, we observed two cases of diabetes mellitus type 2. Anencephaly has also been associated with twinning,^15^ but we did not find any cases of twins in our sample. Anencephaly has also been found as part of the clinical spectrum of other conditions, including some chromosomal abnormalities such as trisomy 18 (Edwards syndrome).^16^ In our sample, all four fetuses who underwent karyotyping showed a normal chromosomal constitution (Table 2). Three fetuses in our sample had a family history of neural tube defects. Moreover, it was noteworthy that all the fetuses presented a final diagnosis of anencephaly. This information could be useful in evaluating suspected cases of anencephaly, since positive findings of a family history of neural tube defects may favor a diagnosis of anencephaly.

The maternal complications reported in pregnancies of anencephalic fetuses include polyhydramnios, dysfunctional labor and prolonged inducement.^17^ In our sample, we observed one case of polyhydramnios among the 22 fetuses with a final diagnosis of anencephaly. It is also noteworthy that the mothers in our sample were very often of young age (less than 20 years) (37.9%). This feature has been correlated with anencephaly^7^ (Tables 1 and 2).

The differential diagnosis of anencephaly includes a number of conditions involving the skull vault and base. An amniotic band is proposed as the cause of a variety of congenital anomalies, which include abnormalities of the skull and face,^18^ and this was observed in two fetuses of our sample. Interestingly, young maternal age, which is a feature related to anencephaly, has also been associated with amniotic bands.^19^ Both mothers of the fetuses with amniotic bands were less than 18 years of age (Table 2).

Acrania, as observed in one fetus of our sample (case 24), is a rare congenital anomaly in which the flat bones of the skull are partially or completely absent, with full but abnormal development of the cerebral hemispheres.^20^^,^^21^ Three-dimensional ultrasound may contribute towards early detection of fetal acrania.^20^ Cases of acrania in which an amniotic band is present and the cephalic pole adheres to the placenta, as seen in our case 24, has also been termed limb-body wall complex.^21^

Fetal brain disruption sequence is considered to be a rare cause of extreme microcephaly. Early death, as observed in our fetuses, is common. Some authors have suggested that different forms of vascular injury to the fetal brain can produce the abnormalities observed in fetal brain disruption sequence.^22^ Some patients may present microhydranencephaly, with complete absence of cerebral hemispheres, as observed in our two fetuses. Microhydranencephaly has also been associated with an autosomal recessive pattern of inheritance and mutations involving the gene *NDE1*.^23^

Merocrania, a defect observed in one fetus of our sample, refers to absence of the skull with the exception of the occipital bone. The brain parenchyma is severely dysmorphic and covered with a thin membrane. Merocrania results from a failure of migration of the mesenchyme under the ectoderm, and associated anomalies such as heart defects are common.^24^

In our sample, we found one case of holoprosencephaly, which is a malformation characterized by an abnormality of separation of the cerebral hemispheres. Most patients present chromosomal abnormalities or syndromic forms of holoprosencephaly.^25^ In our case, we did not observe any presence of additional malformations. However, this fetus did not undergo karyotype evaluation.

Genetic counseling on the risk of recurrence of fetal malformations is dependent on an accurate diagnosis. After a pregnancy consisting of a case of anencephaly, the risk of recurrence in subsequent pregnancies is 2 to 5%. It is important to note that when anencephaly is part of the spectrum of a syndrome, the risk of recurrence will be applied to the syndrome itself. For example, in trisomy 18 cases, recurrence is considered extremely rare, unlike what is seen with anencephaly.^26^ The risk of recurrence of acrania appears to be low.^27^ Regarding merocrania, its pathogenesis is not clearly understood. Folic acid supplementation is known to reduce recurrences of neural tube defects, including anencephaly, by more than 50%.^1^^,^^2^^,^^3^ However, it was noteworthy in our sample that out of the nine pregnant women for whom data on folic acid supplementation was reported, only five received supplementation. Furthermore, all of these pregnant women used folic acid in the wrong way: they started using it after the second month of pregnancy, whereas closure of the neural tube takes place during the first month.

Most children with anencephaly die in utero, and those who are born alive usually die within the first week of life, as observed in our study. No fetal intervention is possible in such cases, and treatment after birth consists only of support.^28^ It was noteworthy in our study that the fetuses with differential diagnoses of conditions other than anencephaly were also quite serious cases, and the observed length of survival was similar to that of the cases of anencephaly.

Termination of pregnancy in a case of an anencephalic fetus was recently authorized by the Federal Supreme Court of Brazil. In our sample, we identified 11 cases of anencephaly that underwent legal termination of pregnancy and, in one of them, this occurred after the implementation of the new law. The timing of the termination was usually somewhat late, around the 24^th^ week of gestation (Figure 1), and this may reflect a delay in the diagnosis. It is important to note that in cases of fetuses with severe abnormalities that are part of the differential diagnoses of anencephaly, termination of pregnancy is not permitted by Brazilian law.

## CONCLUSIONS

Different conditions involving the cranial vault may be confused with anencephaly, as seen in our sample. The diagnosis of anencephaly is usually made during the second trimester of pregnancy, around the 20^th^ week. Factors associated with anencephaly include young maternal age and pregestational diabetes mellitus. A family history of neural tube defects could be a useful finding in evaluating suspected cases of anencephaly. Fetuses with a diagnosis of anencephaly, or even a suspected case of this, seem to have a poor prognosis. In our sample, all of them died during pregnancy or soon after birth. Folic acid supplementation in the maternal diet seems to be insufficient, since only a few pregnant women reported using it, and they used it erroneously.
